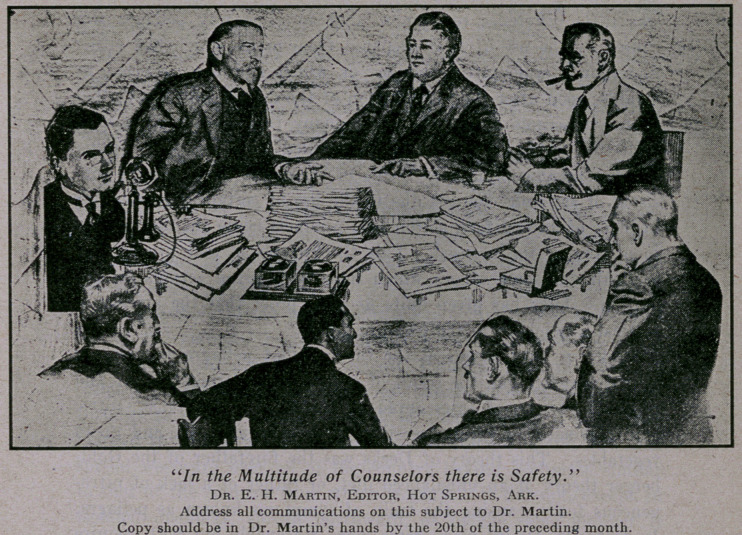# Theories as to Pellagra

**Published:** 1916-11

**Authors:** 


### PELLAGRA FORUM.

*"In the Multitude of Counselors there is Safety."*
DR. E. H. MARTIN, EDITOR, HOT SPRINGS, ARK.
Address all communications on this subject to Dr. Martin.
Copy should be in Dr. Martin's hands by the 20th of the preceding month.

### Theories as to Pellagra.

In no other disease do we find such diverse experiences reported and opinions announced as in pellagra. We should study these various experiences and see if they can be reconciled sufficiently to point to facts which will add to our common fund of knowledge on the subject.

First came the spoiled maize theory.

One hundred and fifty years ago proclamations were made by Italian authorities against the sale or use of spoiled corn. In later years Italian legislation on the same subject has been even more positive and these laws are still in operation. We cannot pass lightly over such experience of centuries in considering a disease which was new to us a decade ago.

It is true that the scientists of Italy have differed widely as to how spoiled corn might cause the disease, one school claiming it due to systemic poisoning from the moulds on spoiled maize acting as drugs, chronic ergotism being a parallel example; other groups of observers seeing in some of these corn parasites the specific organism of pellagra with which the ingestion of spoiled corn merely infected the patient. But for two centuries there was little difference of opinion that in some way pellagra was caused by the use of food prepared from maize which had either undergone fermentative changes or which had been affected by moulds; soon after the introduction of the disease into the United States it became apparent that in many the physical facts were such that we could not accept the spoiled corn theory and other theories became prominent. One of these was that pellagra consisted of systemic changes due to the use of certain vegetable oils in place of animal fats, cotton seed products being the most prominent among the accused. This theory was as well proven theoretically as some others which have been given serious consideration. Jelks has given quite as good proof that the amoeba is at least a neighbor to the pellagra germ.

Deeks in 1912 advanced the theory "that pellagra was due to an autointoxication, the result of corbohydrate fermentation," and reported a series of cases cured by the use of fruit juices, nitric acid and a carbohydrate-free diet of meat, eggs, milk and green vegetables. This theory of Deeks was the forefather of the Goldberger theory of an unbalanced diet, or rather of a lack of nitrogeneous foods, causing the train of symptoms known as pellagra. In fact, Dr. Deeks' view seems more reasonable but both require the same diet.

The disease was thus classed as entirely nutritional, and beriberi and scurvy were compared as kindred nutritional diseases.

Another theory advanced by a group of observers in Europe and backed by Purdue in America proves to the satisfaction of those advancing it that the disease is not due to any of these nutritional causes at all, but to colloidal silica present in the drinking water, and that it is readily cured by calcium or other alkalies.

The believers in a specific organism as the infectious cause of the disease have given us as many logical arguments in support of theories of the spread of the disease by insects, as those above mentioned.

Briefly, no one of these theories has been acceptable to the common sense of the profession at large. One of them may be true, all of them cannot be true, none of them seem to all of us to be true.

The treatment of the disease presents as many different cures claimed to be successful. Dr. Deeks cures by feeding the patients with fruit juices and a carbohydrate-free diet.

Dr. Goldberger cures whole institution-fuls of patients with milk, meat, eggs and beans, also limiting the carbohydrates.

Dr. Purdue promises universally good results by dumping limestone into wells and cisterns. Others obtain strikingly successful results by administering the arsenical preparations. And Dr. Dyer needs nothing specific except quinine.

Let us not err in throwing aside any of these theories or results. They are all honest and all worthy of consideration. Taken without consideration they may seem irreconcilable, but when all are considered deductively they may piece out a mosaic which will pave the way to the truth. For the truth on this subject we do not yet know.

If all of the evidence were submitted to a jury, not of physicians but of the patients' peers, the attorney for food products would thus sum up the case: The accused, Pellagra, lived in a foreign country for more than two centuries that we know of without disturbing us. After being brought to this country it first gave marked trouble on the coast nearest its former domicile. It then spread westward over all of the country having a climate similar to that of the country from which it came. It did not, except in isolated instances, spread northward, as in the same manner it had failed to spread northward in the continent from which it came. The verdict of that jury would be that the cause of pellagra is something alive and that its living cause prefers a moderately warm climate.

## Figures and Tables

**Figure f1:**